# ShapeGTB: the role of local DNA shape in prioritization of functional variants in human promoters with machine learning

**DOI:** 10.7717/peerj.5742

**Published:** 2018-11-29

**Authors:** Maja Malkowska, Julian Zubek, Dariusz Plewczynski, Lucjan S. Wyrwicz

**Affiliations:** 1 Laboratory of Bioinformatics and Biostatistics, Maria Sklodowska-Curie Memorial Cancer Centre and Institute of Oncology, Warsaw, Poland; 2 Laboratory of Functional and Structural Genomics, Centre of New Technologies, University of Warsaw, Warsaw, Poland; 3 Faculty of Mathematics and Information Science, Warsaw University of Technology, Warsaw, Poland

**Keywords:** Single-nucleotide polymorphism, DNA shape, DNA sequence variation, Promoter, Variant prioritization, Machine learning

## Abstract

**Motivation:**

The identification of functional sequence variations in regulatory DNA regions is one of the major challenges of modern genetics. Here, we report results of a combined multifactor analysis of properties characterizing functional sequence variants located in promoter regions of genes.

**Results:**

We demonstrate that GC-content of the local sequence fragments and local DNA shape features play significant role in prioritization of functional variants and outscore features related to histone modifications, transcription factors binding sites, or evolutionary conservation descriptors. Those observations allowed us to build specialized machine learning classifier identifying functional single nucleotide polymorphisms within promoter regions—ShapeGTB. We compared our method with more general tools predicting pathogenicity of all non-coding variants. ShapeGTB outperformed them by a wide margin (average precision 0.93 vs. 0.47–0.55). On the external validation set based on ClinVar database it displayed worse performance but was still competitive with other methods (average precision 0.47 vs. 0.23–0.42). Such results suggest unique characteristics of mutations located within promoter regions and are a promising signal for the development of more accurate variant prioritization tools in the future.

## Introduction

The concept of personalized medicine has made the functional annotation of genomic variations one of the major goals of human genetics. The research inquiries are done both at individual level of low-throughput methods and large-scale population studies. The results of genome-wide association studies of complex human traits have exposed enrichment for variations in the regulatory elements, such as promoters, enhancers, insulators, or intergenic regions. Although about 90% of single nucleotide polymorphisms (SNPs) are located in non-coding regions of human genome, the knowledge about their role in pathology of diseases is limited. In this article, we propose a method for functional prioritization of variants in human promoters, which represent around 1% of all SNPs identified by the 1000 Genomes Project (Ignatieva et al., 2014).

In recent years, several computational methods have been developed to address the challenging task of noncoding variants annotation. These methods differ in the adopted algorithms and utilized data. The main three approaches used by currently available tools are: functional annotations, sequence homology analysis, and machine learning models integrating information from both sources. In particular, the third integrating machine learning approach is worth investigating. The last decade has brought dramatic progress in application of machine learning algorithms in computational biology. Their versatile predictions have been utilized to link noncoding variations properties to their functional nature by that is, genome-wide annotation of variants (Ritchie et al., 2014), combined annotation-dependent depletion (CADD) (Kircher et al., 2014), deleterious annotation of genetic variants using neural networks (DANN) (Quang, Chen & Xie, 2015), FATHMM-MKL (Shihab et al., 2015), deltaSVM (Lee et al., 2015), DeepSEA (Zhou & Troyanskaya, 2015).

Promoters are one of the key regulatory elements of transcription initiation. Several resources indicate that promoter regions show distinct structural constrains when compared with non-promoters (Kanhere & Bansal, 2005; Goni et al., 2007; Morey et al., 2011; Gan, Guan & Zhou, 2012). The analysis by Freeman et al. (2014) shows that the sequence-dependent shape of DNA encodes histone affinity and dominates molecular recognition in the problem of nucleosome positioning. Since various DNA sequences can encode similar shapes (Gardiner et al., 2004; Greenbaum, Pang & Tullius, 2007), correlation between DNA shape descriptors, and biological functions becomes an interesting problem to investigate.

The development of DNAshape web server by Zhou et al. (2013) allowed analyzing DNA structural features on a genomic scale. The method computes four DNA shape features: minor groove width (MGW), roll (Roll), propeller twist (ProT), and helix twist (HelT). Recent studies have showed that combining DNA sequence with DNA local shape improves the prediction accuracy of transcription binding sites in vitro (Rohs et al., 2009; Dror et al., 2014). Here, we address the question of the usefulness of such data in predicting functional effects of sequence variations in promoter regions of genes. We are convinced that the DNA shape features may largely contribute to solving a demanding problem of regulatory variants interpretation and assessment of their effects on disease pathology.

To test this hypothesis and demonstrate its applicability, we trained a machine learning classifier, which uses local shape to predict functional prioritization of promoter sites. In this paper, we compare structural predictor’s performance with sequence-based methods, and analyze in detail the statistical relevance of different types of features characterizing DNA molecule.

In the light of the unique promoter characteristics, inclusive GC distribution (Lenhard, Sandelin & Carninci, 2012; Andersson et al., 2014), transcription factor binding site composition (Rada-Iglesias et al., 2011; Shen et al., 2012; Thurman et al., 2012), and unique chromatin signatures (Heintzman et al., 2007; Hon, Hawkins & Ren, 2009), we focused our analysis on the regions located upstream of the transcription start site. To our best knowledge, previously developed methods have not aimed the variant prioritization in promoter regions by local DNA shape features but rather focused on non-coding sequence variations without acknowledging genomic region.

## Materials and Methods

### Datasets

To obtain the positive dataset we used single-nucleotide variants (SNVs) annotated as regulatory mutations in The Human Gene Mutation Database (HGMD^®^) professional version (release 2016.2) within five kilobases (kb) upstream from the annotated transcription start sites (TSS) and provided sequences (Stenson et al., 2014). The total number of experimentally validated disease-related variants in our dataset is equal to 1,772. The control dataset contains SNVs from the 1000 Genomes Project (The 1000 Genomes Project Consortium et al., 2015) with a global minor allele frequency ≥1%. The overlapping elements of both sets were removed. Only variants lying within five kb upstream of TSS were selected for further analysis (Rosenbloom et al., 2015). The sequences of neutral motifs (not associated with disease phenotype) were retrieved from Ensembl with BioMart (Kinsella et al., 2011). The total number of negative examples in our dataset is equal to 3,806. We ensured that positive and negative motif sets are matched in their basic properties (Kolmogorov–Smirnov two sample test results for GC-content distributions are as follows *D*-statistic = 0.02, *p*-value = 0.48, null hypothesis of identical distributions retained). Distributions of TSS distances in the two sets differed, but we made sure that it does not affect obtained results (see Supplementary Material S5).

### Machine learning pipeline

We split the available data into training and test sets randomly keeping the ratio 8:2. Full training set contained 1,417 positives and 3,045 negatives, full test set contained 355 positives and 761 negatives. Training set was used to build feature ranking, train classifiers, and optimize their parameters, while test set was left for final validation and for comparison with other prediction methods. To validate our methods internally on the training set we used a cross-validation strategy in which in each fold SNPs from a single chromosome formed test set and SNPs from other chromosomes formed training set. This eliminated possibility of overfitting during parameter tuning and feature selection procedures, and additionally demonstrated whether our method generalizes across different chromosomes.

We applied the Monte Carlo feature selection (MCFS) algorithm (Draminski et al., 2008) to perform feature importance ranking. It is a universal feature selection strategy combining random subspace methods with decision trees. A random subset of the original features is drawn in each iteration of the algorithm and an equivalent of random forest is induced using the selected variables. Feature importance ranking is constructed based on all induced trees. Additionally, meaningful interdependencies between features are discovered by calculating how often two features are used together to predict the class value. MCFS aims at finding all features relevant for the classification task, and it guarantees that with sufficient number of iterations all features can be tested. Following general guidelines by the authors of the algorithm, we set the number of iterations to 1,000 and the subset of original features considered in each iteration to 0.25.

In the classification task gradient tree boosting was used (GTB)—a popular tree-based ensemble algorithm (Friedman & Meulman, 2003). It is known to perform very well in many domains, often outperforming methods such as random forest, support vector machines or neural networks (Sheridan et al., 2016; Ladds et al., 2016; Babajide Mustapha & Saeed, 2016). The key idea behind GTB is to build trees sequentially, training a tree at each step to explain the prediction error made by the combination of existing trees. Usually the trees are regularized to prevent overfitting. We used the state-of-the-art implementation provided by XGBoost library (Chen & Guestrin, 2016). Through cross-validation performed on the training set we selected optimal parameter values (number of trees—300, maximal tree depth—8, learning rate—0.1).

### Comparison with existing approaches

Presently, the field of prediction and prioritization of human noncoding regulatory variants still lacks a large, independent and publicly available gold-standard dataset for training, testing, and validating existing in silico approaches. The comparison of our method to the current state-of-the-art methods is hampered even further by different aims and objectives. To our best knowledge all available tools were designed for genome-wide, regulatory variants prioritization and there are no computational methods focused on promoter regions. Nonetheless, we compared performance of our algorithm with other tools on our own hold-out test set and on independent high-quality data from ClinVar database (January 5, 2017 release) after excluding variants present in our training data (Landrum et al., 2016). Our hold-out test set contained 355 positives from HGMD and 761 negative examples from 1000 Genomes Project. External validation set contained 32 positive examples labeled as pathogenic in ClinVar database and 761 negative examples from 1000 Genomes Project (not present in our train set).

### Features groups

We used the following feature groups to annotate each SNV in our pathogenic and control datasets (more detailed description can be found in Supplementary Material S1 and S4):

1. *DNA sequence* (52 variables): 9-nt sequence motifs centered on the mutated nucleotide. The sequence was encoded using 4-bits binary coding. Additional 12 binary (4-nt by three mutations) variables indicated what type of mutation occurred (e.g., A → C, G → T, etc.).
2. *Local DNA shape features* (88 variables): HelT, MGW, ProT, roll values in span of 9 nt. Differences (*_diff*) between reference and mutated scores were added as additional features (Chiu et al., 2016).
3. *GC-content* (8 variables): *GC-content* in span of 7- and 9-nt for reference and mutated sequences separately. Differences between the reference and mutated scores were added as additional features.
4. *Histone modifications* (38 variables): ChIP-seq data for histone 3 lysine 9 acetylation (H3K9ac) and histone 3 lysine 4 trimethylation (H3K4me3) across 16 cell lines from ENCODE (Ram et al., 2011). For H3K9ac, H3K4me3, or either modification mean values over all cell lines and binary variables indicating modifications in any cell line were added.
5. *Transcription Factor Binding Sites* (12 variables): TFBS ChIP-seq clusters (V3) from ENCODE data retrieving binding sites of top 10 TFs with the highest binding site coverage. Mean value over all TFs and 0–1 indicator of any TF occurrence were added in addition (ENCODE Project Consortium, 2012).
6. *Transcription factor binding disruption* (one variable): *P*-value of disrupting putative strongest transcription factor binding site due to mutation was calculated with Annotation of Regulatory Variants using Integrated Networks (ARVIN) algorithm (Gao et al., 2018) using Cis-BP database (Weirauch et al., 2014).
7. *Maximum transcription factor binding log-odds ratio score* (one variable): Maximum TF binding log-odds ratio score for reference and mutated sequences among scores calculated with ARVIN algorithm (Gao et al., 2018, Weirauch et al., 2014).
8. *DNase I hypersensitivity* (one variable): ENCODE DNase clusters (V3) from 125 cell line types (John et al., 2011; Thurman et al., 2012; Rosenbloom et al., 2013).
9. *Evolutionary conservation* (10 variables):
  - GERP ++: Genomic Evolutionary Rate Profiling scores (Davydov et al., 2010).
  - PhastCons: PhastCons conservation score by vtools (San Lucas et al., 2012).
  - *Z*-score: recalculated *Z*-score values defined in our previous work (Wyrwicz et al., 2007) on whole genome human–mouse alignments (genome builds hg19 and mm9 (Chiaromonte, Yap & Miller, 2002; Kent et al., 2003; Schwartz et al., 2003) from UCSC Genome Browser (Kent et al., 2002) for the reference and mutated sequence and for window length 7 and 9. Differences of *Z*-scores for the reference and mutated sequence were added.
10. *Dinucleotide content* (16 variables): Observed vs. expected frequencies of 16 possible pairs of nucleotides appearing in the short sequence motif.

## Results

### Feature importance

From MCFS we obtained the ranking of all 227 features according to their relative importance in the classification problem. Each feature group contained multiple individual features with different ranks in the overall ranking. In the context of machine learning task, usefulness of a particular group should be determined by the best performing features from this group.

**Figure 1:**
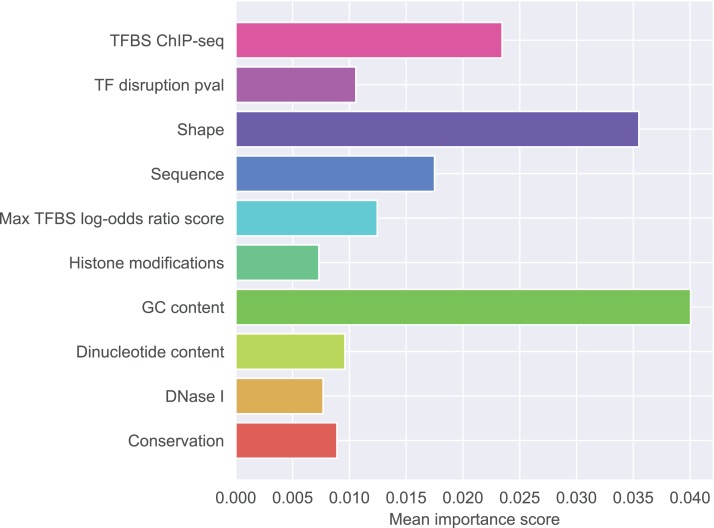
Mean importance of five best scoring features in each feature group.

Figure 1 presents detailed feature ranking including all features from each group. Generally, features that contribute to the correct classification mostly belong to GC-content group, shape group, and sequence group. Other feature groups were of lesser importance (the full ranking is included as Supplementary Material S2, feature names glossary as Supplementary Material S4). The most important feature was the difference in GC-content between the reference and the mutated sequence fragment (rank 1). Features describing raw nucleotide sequence and dinucleotide content appeared in the middle of the ranking. Among the shape features, those describing the closest neighborhood of the mutated nucleotide were the most important. This is not surprising because differences in shape are expected to have local effects on DNA properties. Among the shape features attributes concerning ProT were ranked as the most important, attributes concerning HelT and roll followed, and attributes concerning MGW occurred lower in the ranking. What is notable, most of the features appearing among the top 20 concerned differences in shape properties between SNP and wild type. Features derived from transcription factors were less important than sequence-based features. Histone modifications, conservation scores and DNase I hypersensitivity score were not identified as particularly informative features.

To investigate the role of individual features we calculated Welch’s *t*-score capturing the relationship between particular feature and class value. The decrease of GC-content between the reference and the mutated sequence correlated negatively with functionality (*t*-score −8.2088 for decrease for motif length 7, *t*-score −11.3710 for decrease for motif length 9), while the increase of ProT value correlated positively (*t*-score 9.7417 for increase immediately before the modified nucleotide, *t*-score 5.5047 for increase immediately after the modified nucleotide).

**Figure 2:**
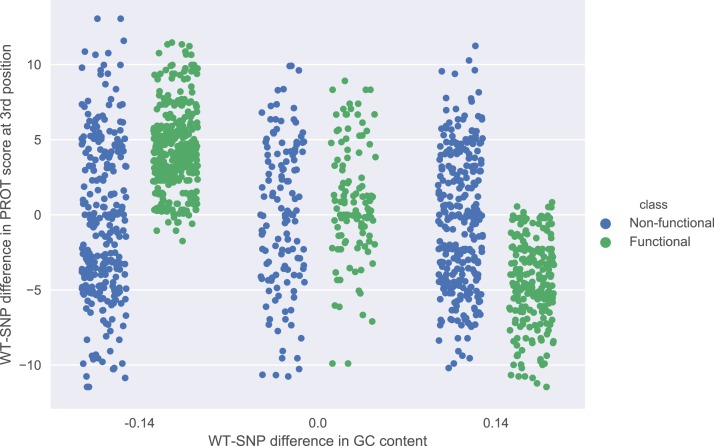
Joint distributions of the two most important features in the two classes. WT-SNP difference corresponds to difference of scores between reference (wild type) and mutated (SNP) variants.

**Figure 3:**
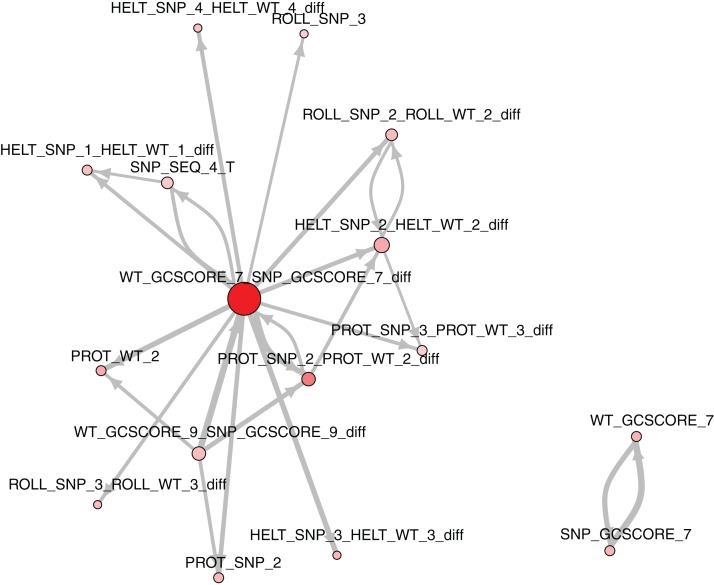
The strongest feature interdependencies.

The role of each feature in a classification task lies not only in its correlation with class value, but also in how well it complements with other features. For example, Fig. 2 presents joint distributions of the two most important features in the two classes (difference of GC-content between the reference and the mutated sequence, difference of ProT at the 3rd position between mutated variant and wild type). For non-functional SNPs the features are uncorrelated, but there is a visible negative correlation for functional SNPs. MCFS allows studying that kind of dependencies through its interdependency discovery function. The full list of feature interdependencies and their relative strength is included as Supplementary Material S3. Figure 3 presents graph of the strongest interdependencies among the top selected features (GCSCORE, GC composition; SEQ, sequence feature; ROLL, roll; HELT, helix twist; PROT, propeller twist). Difference in GC-content acts as a central hub and interacts strongly with all groups of shape features except MGW. The simplified intuition is that functional SNPs should increase GC-content of the motif, and at the same time increase rotation of the DNA strand accordingly.

### Classifier performance

Obtained feature ranking suggests that a large portion of information is contained in features derived from the DNA sequence, and features describing evolutionary conservation and functional properties play less significant role. To verify this hypothesis, we performed a cross-validation experiment (with folds determined by chromosomes) on the train set by training GTB classifier on different combinations of feature groups. Calculated values of multiple performance measures are presented in Table 1.

**Table 1:** Cross-validation classification results for different feature groups on TSS-balanced data set.

	AUC	AUC_std	Accuracy	Accuracy_std	F1	F1_std	Precision	Precision_std	Recall	Recall_std	size
All	0.9764	0.0133	0.9258	0.0247	0.8803	0.0456	0.8840	0.0643	0.8792	0.0480	227.0
Best 25	0.9243	0.0345	0.8449	0.0418	0.7551	0.0785	0.7456	0.1079	0.7713	0.0710	25.0
Sequence	0.5555	0.0473	0.6162	0.0584	0.3170	0.0416	0.3766	0.0878	0.2834	0.0453	52.0
GC-content	0.7765	0.0525	0.7051	0.0626	0.4934	0.0634	0.5560	0.1054	0.4546	0.0713	8.0
Shape	0.5571	0.0566	0.6251	0.0690	0.2546	0.0597	0.3574	0.0994	0.2039	0.0551	88.0
Conservation	0.5440	0.0416	0.6569	0.0522	0.2693	0.0764	0.4313	0.1547	0.2003	0.0545	10.0
TFBS ChIP-seq	0.5255	0.0482	0.6674	0.0755	0.2416	0.0707	0.4722	0.1589	0.1683	0.0550	12.0
Histone modifications	0.5664	0.0641	0.6270	0.0690	0.3342	0.0702	0.3987	0.1069	0.2994	0.0844	38.0
DNase I	0.5846	0.0622	0.6662	0.0817	0.1474	0.0674	0.4088	0.1921	0.0914	0.0431	1.0
Dinucleotide content	0.5205	0.0615	0.6211	0.0614	0.2354	0.0798	0.3407	0.1323	0.1858	0.0647	16.0
Max TFBS log-odds ratio score + TF disruption pval	0.5141	0.0613	0.6773	0.0824	0.0364	0.0381	0.3812	0.3618	0.0193	0.0205	2.0
Sequence + GC-content	0.7689	0.0404	0.6997	0.0465	0.5029	0.0578	0.5426	0.1159	0.4816	0.0477	60.0
Shape + GC-content	0.9175	0.0313	0.8395	0.0333	0.7399	0.0627	0.7557	0.1052	0.7332	0.0583	96.0
Sequence + GC-content + Shape	0.9787	0.0140	0.9446	0.0208	0.9124	0.0381	0.8894	0.0616	0.9400	0.0437	148.0
Sequence + GC-content + Shape + TF disruption pval	0.9787	0.0132	0.9471	0.0231	0.9161	0.0400	0.8899	0.0624	0.9468	0.0401	149.0
Sequence + GC-content + Shape + TF disruption pval + Max TFBS log-odds ratio score	0.9782	0.0139	0.9442	0.0189	0.9118	0.0318	0.8933	0.0595	0.9346	0.0374	150.0
Sequence + GC-content + TFBS ChIP-seq	0.7902	0.0332	0.7206	0.0410	0.5252	0.0614	0.5698	0.0934	0.4933	0.0616	72.0
Sequence + GC-content + Histone modifications	0.7981	0.0426	0.7249	0.0464	0.5359	0.0656	0.5882	0.1170	0.5054	0.0664	98.0

Classifier based on all available features performed better than the classifier using only 25 best ranked features. Among individual feature groups GC-content produced classifier with the largest AUC ROC (0.78). Combining GC-content with shape features and sequence features allowed achieving AUC ROC 0.98. No other combinations of features performed better. These results show that shape features are more meaningful when combined with another feature. In further experiments classifier trained on sequence, shape, and GC-content was used. We named this classifier ShapeGTB.

**Figure 4:**
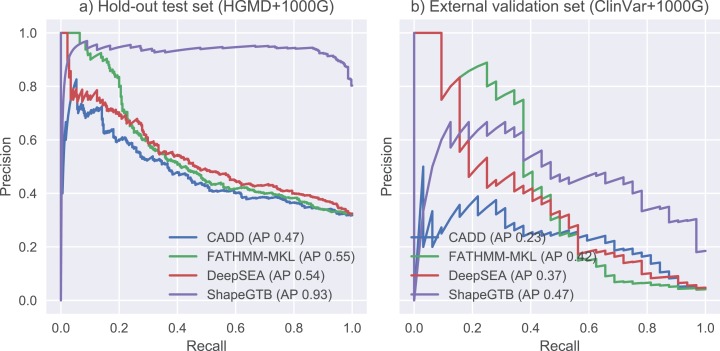
Precision-recall curves for different classifiers. Results are given for hold-out test set (A) and an external validation set based on ClinVar data (B).

**Figure 5:**
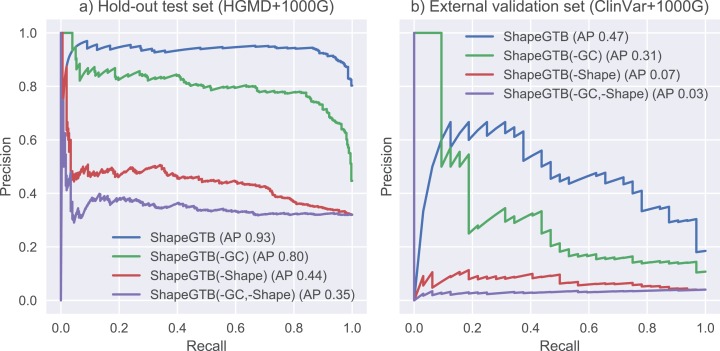
Precision-recall curves for variants of ShapeGTB in which feature vectors from specific feature groups were permuted (effectively reducing their usefulness). -GC corresponds to classifier with GC-derived features permuted, -Shape corresponds to classifier. Results are given for hold-out test set (A) and an external validation set based on ClinVar data (B).

We compared final ShapeGTB classifier with more general SNP prioritization methods, which did not focus specifically on promoter regions: CADD, FATHMM-MKL, and DeepSEA. Figure 4 present precision-recall curves calculated on the hold-out test set constructed from our data (HGMD and 1000 Genomes Project) and for smaller experimental dataset (ClinVar and 1000 Genomes Project). Area under precision-recall curve can be interpreted as average precision (AP), and is an aggregated measure of classifier performance. It is preferred over AUC ROC when the problem is characterized by a large class imbalance. On the hold-out test set, ShapeGTB outperformed general-purpose methods by a large margin (AP 0.93 vs. 0.47–0.55). On the external validation set, the ShapeGTB aggregated performance was comparable with FATHMM-MKL (AP 0.47 vs. AP 0.42). However, shapes of precision-recall curves for those methods were very different: FATHMM-MKL displayed high precision only for small subset of examples, while ShapeGTB precision was relatively stable even for large values of recall. Differences between results obtained for the two datasets suggest that ClinVar-derived positives have different characteristics and pose a greater challenge. We speculated that the gap between ShapeGTB and reference tools on the hold-out test is due to inclusion of shape features and their interactions with GC-content. To verify this, we randomly permuted these features in our test set and evaluated performance of ShapeGTB again on permuted data sets. AP of ShapeGTB with GC-derived features permuted was 0.80, with shape-derived features permuted 0.44, and with both kinds of features permuted 0.35 (Fig. 5). This once more corroborates the hypothesis that shape features together with GC-content provide important information for distinguishing functional SNPs in our data set.

## Discussion and Conclusions

Here, we report the influence of the combined multifactor analysis of DNA shape and other descriptors in prediction of functional effect of promoter variants. Previously, Parker et al. (2009) has demonstrated that the nucleotide alternations can significantly affect the DNA structure causing changes in protein binding affinity and phenotype. From our analysis, it is clear that changes in the geometry of DNA molecule are important features for the task of prioritization of functional regulatory variants within promoter regions. General conclusions that can be drawn from our study are as follows: (a) shape features work very locally, what is important is what happens in the closest neighborhood of the mutated nucleotide, (b) DNA chain rotations are more important than MGW, (c) differences of properties of the mutated variant and the reference motif are the most meaningful. This picture is inherently complicated with the presence of feature interdependencies—mostly between GC-content and shape features. It is impossible to make predictions based on DNA shape alone, it is meaningful only with respect to the sequence content.

Interestingly, in our method the most informative indicator of variant functional impact is whether the introduced nucleotide changes the GC-content. The GC composition has been previously linked to DNA thermostability, bendability, and potential for conformational transition between B- and Z-forms, that relate to chromatin accessibility (Vinogradov, 2003). The instances of GC-rich sequence motifs have been shown to play an important role in transcription regulation through their connection with nucleosome occupancy and TF binding (Peckham et al., 2007; Wang et al., 2012). In our opinion, high rank of GC-ratio derivatives is a result of promoter properties, which distinguish it from other regulatory elements (Lenhard, Sandelin & Carninci, 2012; Andersson et al., 2014). GC-ratio may not be highly ranked if similar analysis would be performed on other regulatory elements, which are not associated with promoter regions (e.g., splicing elements or insulators).

There is a vast amount of literature on complex networks of relations between nucleotide types and various shape attributes (Yoon et al., 1988; Florquin et al., 2005; Rohs, Sklenar & Shakked, 2005; Samanta et al., 2009). For instance, the distribution of water around the minor groove shows specificity to the DNA sequence as the availability of the hydrogen bond forming atoms changes. Variation in DNA sequence may affect DNA flexibility by influencing the magnitude of ProT. Specific base pairs combinations have different electrostatic potentials and prefer specific stacking geometry (Samanta et al., 2009). The results of Tillo & Hughes (2009) have highlighted that GC-ratio influences nearly all aspects of DNA structure. The most pronounced dependency has been observed between GC-ratio and ProT (Ponomarenko et al., 1999). Deb et al. (1987) previously reported the effect of an A/T base pair replacement by a G/C base pair on narrowing of minor grows through negative propeller twisting. This pair has also been rated high in our feature interdependencies ranking. To sum up, it appears that only a specific configuration of local structural feature values can meet the requirements of a functional genomic element and that causative mutation substantially disrupt it consensus.

The data derived from ChIP-seq experiments and DNaseI hypersensitivity assays have relatively low resolution generally ranging from 200 to 8 kbp (Park, 2009; Pique-Regi et al., 2011; ENCODE Project Consortium, 2012). Our analysis shows that histone modification and TFBS ChIP-seq peaks along with TF disruption *p*-value and DNaseI hypersensitivity data, being used in genome-wide setting, have no discriminative power for promoter region sequence variations. This is especially true for TSS-balanced version of our data sets (Supplementary Material S5). It is important to stress that features based on histone modifications and TFBS have different meaning than those derived directly from DNA sequence and shape. The former may represent statistical relationships connected with high-level functioning of the organism, while the latter may correspond to low-level binding mechanisms and biophysical properties of the DNA. Our method is able to make successful predictions using only low-level features, which may inform the study of low-level mechanisms behind functional SNP mutations.

There is a strong need in the field for entirely independent, high-quality collection of regulatory elements variants categorized by type of non-coding sequence and functional status. Such collection would allow constructing reliable tests sets to validate and compare available methods. According to Li & Wang (2017) analysis, human genetic variants databases such as HGMD and ClinVar contain contradictory entries and incorrectly categorized variants due to the lack of primary review of evidence.

In our experiments, our method outperformed significantly the reference tools on our own dataset, and exhibited better recall on external dataset. However, caution is required in drawing final conclusions from the comparison. Our model targeted promoter regions specifically, while the other tools were trained on larger subsets of non-coding regions. It is also possible that our validation set, at least partially, overlapped with training sets used by other algorithms. We believe that the main reason behind good performance of ShapeGTB is the inclusion of shape features. Without them the expected performance is on par with the other methods (AP 0.44 on hold-out test set).

In summary, we demonstrated that the local shape features of DNA surrounding single nucleotide coupled with the GC-content and sequence composition are sufficient for single nucleotide variant prioritization within promoter regions of human genes. Our results additionally confirmed the interdependencies between alternations in the GC-content and local DNA shape features. Given that the shape vectors implicitly reflect electrostatics, base stacking, hydration profiles (Przytycka & Levens, 2015), including DNA shape into model results in functional reduction of the number of features and therefore a great simplification of the method. We believe that local DNA shape features carry a vast amount of information and their applicability should be investigated further. In the future, we plan to extend our analysis on all types of regulatory elements in non-coding regions of human genome.

## Supplemental Information

10.7717/peerj.5742/supp-1 Supplemental Information 1 Feature groups description.

10.7717/peerj.5742/supp-2 Supplemental Information 2 Features ranking.

10.7717/peerj.5742/supp-3 Supplemental Information 3 Feature interdependencies.

10.7717/peerj.5742/supp-4 Supplemental Information 4 Glossary.

10.7717/peerj.5742/supp-5 Supplemental Information 5 ROC curves for random train-test split.

## Additional Information and Declarations

### Data Availability

The following information was supplied regarding data availability: GitHub: https://github.com/zubekj/ShapeGTB.
