# Supplementary material for: ShapeGTB: the role of local DNA shape in prioritization of functional variants in human promoters with machine learning
Source: PeerJ. 2018 Nov 29;6:e5742. doi: 10.7717/peerj.5742 (PMC6275119; doi:10.7717/peerj.5742)
Supplement: Supplemental Information 1 [file peerj-06-5742-s001.docx]

Supplementary material 1

Features groups

***DNA sequence*** (52 variables):

We observed that the changes in DNA shape introduced by single-site mutations extend up to ± 4-nt, therefore motif sequence was extracted using the spanning window of 9 nucleotide bases centered on the mutated nucleotide. The sequence was encoded using one-hot encoding, i.e. for each window position for binary variables were introduced indicating whether nucleotide at the given position is A, C, G or T. Similarly, 4 binary variables encoded the nucleotide at the central position in the mutated sequence. Additional 12 binary (4-nt by 3 mutations) variables indicated what type of mutation occurred, for example A → C, G → T, etc..

***Local DNA shape features*** (88 variables):

We retrieved helix twist (HelT), minor groove width (MGW), propeller twist (ProT), roll (Roll) values with DNAshapeR tool (Chiu, et al., 2016). HelT and Roll vectors of values had length 6, MGW and ProT length 5. These features were calculated separately for reference and mutated sequences. Additionally, for each position and each shape feature we added difference and product of the reference and the mutated variant as additional features.

***GC-content*** (8 variables):

We calculated fractional GC-content in span of 7 and 9 nucleotide bases for reference and mutated sequences separately with Biopython (Cock, et al., 2009). Distributions of GC-content scores for positive and negative motif sets were similar (the Kolmogorov–Smirnov two sample test results for GC content distributions are as follows D-statistic=0.02, p-value=0.48, null hypothesis of identical distributions retained). Differences and products of the reference and mutated GC-content scores were added as additional and potentially meaningful features.

***Histone modifications*** (38 variables):

We used broad peaks of ChIP-seq data for histone 3 lysine 9 acetylation (H3K9ac) and histone 3 lysine 4 trimethylation (H3K4me3) across 16 cell lines from ENCODE (Ram, et al., 2011). H3K9ac and H3K4me3 are the most common histone modifications associated with active transcription of genes (Nishida, et al., 2006; Guenther, et al., 2007). For H3K9ac, H3K4me3 or either modification mean values over all cell lines and binary variables indicating modification occurrence in any cell line were added.

***Transcription Factor Binding Sites*** (12 variables):

We used TFBS clusters (V3) from ENCODE data retrieving binding sites of top 10 TFs with the highest binding site coverage: CEBPB, CTCF, EP300, FOS, GATA2, JUND, MAX, MYC, POLR2A, RAD21 (Gerstein, et al., 2012; Wang, et al., 2012; Lu, et al., 2015). Mean value over all TFs and 0-1 indicator of any TF occurrence were added in addition.

***Transcription factor binding disruption*** (1 variable):

P-value of disrupting putative strongest transcription factor binding site due to mutation was calculated with Annotation of Regulatory Variants using Integrated Networks (ARVIN) algorithm (Gao, et al., 2018) using Cis-BP database (Weirauch et al., 2014).

***Maximum transcription factor binding log-odds ratio score*** (1 variable):

Maximum TF binding log-odds ratio score for reference and mutated sequences among scores calculated with Annotation of Regulatory Variants using Integrated Networks (ARVIN) algorithm (Gao, et al., 2018) using Cis-BP database (Weirauch et al., 2014). The method computed scores for 266 Transcription factors in total. Their list can be found in Supplementary Material 4.

***DNase I hypersensitivity*** (1 variable):

We used ENCODE DNase clusters (V3) from 125 cell line types (John, et al., 2011; Thurman, et al., 2012; Rosenbloom, et al., 2013).

***Evolutionary conservation*** (10 variables):

a) GERP ++

We used the Genomic Evolutionary Rate Profiling scores (Davydov, et al., 2010).

b) PhastCons

We obtained PhastCons (Siepel, et al., 2005) conservation scoring with the use of vtools (San Lucas, et al., 2012).

c) Z-score

Following our previous work (Wyrwicz, et al., 2007) we recalculated Z-score values on whole genome human–mouse alignments (genome builds hg19 and mm9 (Chiaromonte, et al., 2002; Schwartz, et al., 2003; Kent, et al., 2003) from UCSC Genome Browser (Kent, et al., 2002). We calculated the score separately for the reference and mutated sequence and for window length 7 and 9. Differences and products of Z-scores for the reference and mutated sequence were added. The updated dataset of conservation Z scores for human-mouse promoters is available upon request.

***8. Dinucleotide content*** (16):

Observed vs. expected frequencies of 16 possible pairs of nucleotides appearing in the short sequence motif.
