# Supplementary material for: ShapeGTB: the role of local DNA shape in prioritization of functional variants in human promoters with machine learning
Source: PeerJ. 2018 Nov 29;6:e5742. doi: 10.7717/peerj.5742 (PMC6275119; doi:10.7717/peerj.5742)
Supplement: Supplemental Information 4 [file peerj-06-5742-s004.doc]

Supplementary material 4

**SNP_ID** – variant ID

**CHROM** – chromosome labels

**POS** – position on a chromosome

**TSS_distance** – distance from transcription start site

**DNASE_SCORE** – DNaseI Hypersensitivity Cluster score

**GERP_SCORE** – GERP++ score

**PHASTCONS_SCORE** – PhastCons score

**HELT_SNP_k** – HelT score at position k of variant motif, where k is an integer between 0 and 5 (e.g. HELT_SNP_0 – HelT score at position 0 of variant motif)

**HELT_WT_k** – HelT score at position k of wild-type motif, where k is an integer between 0 and 5 (e.g. HELT_WT_0 – HelT score at position 0 of wild-type motif)

**HELT_SNP_j_HELT_WT_k_diff** – difference between HelT scores at positions j, k (from 0 to 5) between variant and wild-type motif (e.g. HELT_SNP_0_HELT_WT_0_diff – difference between HelT scores at position 0 of variant and wild-type motif)

**HELT_SNP_j_HELT_WT_k_mul** – multiplication of HelT scores at positions j, k (from 0 to 5) for variant and wild-type motif (e.g. HELT_SNP_0_HELT_WT_0_mul – multiplication of HelT scores at position 0 of variant and wild-type motif)

**HIST_SCORE_xy** – Histone modification ChIP-seq peak score, where x is cell line type (A549, Dnd41, Gm12878, H1hesc, Helas3, Hepg2, Hmec, Hsmm, Huvec, K562, Monocd14ro1746, Nha, Nhdfad, Nhek, Nhlf) and y is histone modification (H3k04me or H3k09ac)

**MEAN_HIST_9ac** – mean of all cell line types ChIP-seq peak scores for 9ac

**MEAN_HIST_me3 –** mean of all cell line types ChIP-seq peak scores for me3

**mean_Hist** – mean of all cell line types zzz scores for 9ac and me3

**0_1_Hist –** presence of any type of histone modification in any type of cell line (0 – no, 1 – yes)

**0_1_Hist_9ac** – presence of 9ac modification in any type of cell line (0 – no, 1 – yes)

**0_1_Hist_me3** – presence of me3 modification in any type of cell line (0 – no, 1 – yes)

**MGW_SNP_k** – MGW score at position k of variant motif, where k is an integer between 0 and 4 (e.g. MGW_SNP_0 – MGW score at position 0 of variant motif)

**MGW_WT_k** – MGW score at position k of wild-type motif, where k is an integer between 0 and 4 (e.g. MGW_WT_0 – MGW score at position 0 of wild-type motif)

**MGW_SNP_j_MGW_WT_k_diff** – difference between MGW scores at positions j, k (from 0 to 4) between variant and wild-type motif (e.g. MGW_SNP_0_MGW_WT_0_diff – difference between MGW scores at position 0 of variant and wild-type motif)

**MGW_SNP_j_MGW_WT_k_mul** – multiplication of MGW scores at positions j, k (from 0 to 4) for variant and wild-type motif (e.g. MGW_SNP_0_MGW_WT_0_mul - multiplication of MGW scores at position 0 of variant and wild-type motif)

**PROT_SNP_k** – ProT score at position k of variant motif, where k is an integer between 0 and 4 (e.g. PROT_SNP_0 – ProT score at position 0 of variant motif)

**PROT_WT_k** – ProT score at position k of wild-type motif, where k is an integer between 0 and 4 (e.g. PROT_WT_0 – ProT score at position 0 of wild-type motif)

**PROT_SNP_j_PROT_WT_k_diff** – difference between ProT scores at positions j, k (from 0 to 4) between variant and wild-type motif (e.g. PROT_SNP_0_PROT_WT_0_diff – difference between ProT scores at position 0 of variant and wild-type motif)

**PROT_SNP_j_PROT_WT_k_mul** – multiplication of ProT scores at positions j, k (from 0 to 4) for variant and wild-type motif (e.g. PROT_SNP_0_PROT_WT_0_diff – multiplication of ProT scores at position 0 of variant and wild-type motif)

**ROLL_SNP_k** – Roll score at position k of variant motif, where k is an integer between 0 and 5 (e.g. ROLL_SNP_0 – Roll score at position 0 of variant motif)

**ROLL_WT_k** – Roll score at position k of wild-type motif, where k is an integer between 0 and 5 (e.g. ROLL_WT_0 – Roll score at position 0 of wild-type motif)

**ROLL_SNP_j_ROLL_WT_k_diff** – difference between Roll scores at positions j, k (from 0 to 5) between variant and wild-type motif (e.g. ROLL_SNP_0_ROLL_WT_0_diff – difference between Roll scores at position 0 of variant and wild-type motif)

**ROLL_SNP_j_ROLL_WT_k_mul** – multiplication of Roll scores at positions j, k (from 0 to 5) for variant and wild-type motif (e.g. ROLL_SNP_0_ROLL_WT_0_mul – multiplication of Roll scores at position 0 of variant and wild-type motif)

**SNP_GCSCORE_7** – GC content of 7 nucleotide-long variant motif (values between 0 and 1)

**SNP_GCSCORE_9** – GC content of 9 nucleotide-long variant motif (values between 0 and 1)

**WT_GCSCORE_7** – GC content of 7 nucleotide-long wild-type motif (values between 0 and 1)

**WT_GCSCORE_9** – GC content of 7 nucleotide-long wild-type motif (values between 0 and 1)

**WT_GCSCORE_7_SNP_GCSCORE_7_diff** – difference between GC contents of 7 nucleotide-long wild-type and variant motifs

**WT_GCSCORE_7_SNP_GCSCORE_7_mul** – multiplication of GC contents of 7 nucleotide-long wild-type and variant motifs

**WT_GCSCORE_9_SNP_GCSCORE_9_diff** – difference between GC contents of 9 nucleotide-long wild-type and variant motifs

**WT_GCSCORE_9_SNP_GCSCORE_9_mul** – multiplication of GC contents of 9 nucleotide-long wild-type and variant motifs

**SNP_SEQ_k_n** – presence of nucleotide n at position k of variant motif, where n is adenine(A), thymine (T), cytosine(C) or guanine (G) and k is an integer between 0 and 8 (e.g. SNP_SEQ_0_A – presence of adenine at position 0 of variant motif; 0 – no, 1 – yes)

**WT_SEQ_4_n** – presence of nucleotide n at position 4 of wild-type motif, where n is adenine (A), thymine (T), cytosine(C) or guanine (G) (e.g. WT_SEQ_0_A – presence of adenine at position 0 of wild-type motif; 0 – no, 1 – yes)

**WT_SNP_nm** – presence of mutation n -> m, where n and m are nucleotides: adenine (A), thymine (T), cytosine(C) or guanine (G) (e.g. WT_SNP_AC – presence of mutation A -> C; 0 – no, 1 – yes)

**TFBS_SCORE_x** – Transcription Factor ChIP-seq cluster score, where x is CEBPB, CTCF, EP300, FOS, GATA2, JUND, MAX, MYC, POLR2A or RAD21

**mean_TF** – mean Transcription Factor ChIP-seq cluster score

**0_1_TF** – presence of transcription binding site within motif (0 – no, 1 – yes) (based on Transcription Factor ChIP-seq cluster score)

**TF_disruption** – maximum log-odds ratio score for reference and mutated sequences among scores computed for the following transcription factors: AHRR, AL844527.7,ALX1, ALX3, ALX4, ARID2, ARID3A, ARNTL, ARX, ATF1, ATF2, ATF3, ATF5, BARHL1, BARHL2, BARX1, BATF, BATF3, BBX, BCL6, BCL6B, BHLHE40, BHLHE41, BSX, C11orf9, CDX1, CDX2, CEBPA, CEBPB, CEBPD, CEBPE, CEBPG, CPEB1, CREB1, CREB3L1, CRX, CUX1, CUX2, DLX2, DLX3, DLX4, DLX5, DLX6, DMRT1, DRGX, E2F4, E2F7, E2F8, EBF1, EGR1, EGR2, EGR4, ELF2, EN1, EOMES, ESR1, ESR2, ESRRA, ESRRB, ESRRG, FOSL2, FOXA1, FOXA2, FOXB1, FOXB2, FOXC1, FOXC2, FOXD2, FOXD3, FOXE3, FOXF1, FOXF2, FOXG1, FOXI1, FOXJ2, FOXJ3, FOXK2, FOXL1, FOXO1, FOXO3, FOXO4, FOXO6, FOXP1, FOXS1, FUBP1, GBX2, GCM1, GLI1, GLI2, GLI3, GMEB2, HESX1, HNF1B, HNF4A, HNF4G, HOXC10, HOXC12, HOXD11, HOXD12, HOXD3, HOXD4, HSF1, HSFY2, IKZF1, INSM1, ISL2, ISX, JDP2, JUN, JUNB, KLF9, LBX1, LBX2, LEF1, LHX6, LHX9, LIN54, LMX1A, LMX1B, MAF, MAFA, MAFB, MAFF, MAFK, MEF2A, MEIS1, MEIS2, MEIS3, MEOX2, MLX, MLXIPL, MSX1, MSX2, MTF1, NANOG, NFATC1, NFE2, NFIA, NFIB, NFIX, NKX2-5, NKX2-8, NKX3-1, NOBOX, NR1I2, NR1I3, NR2C1, NR2C2, NR2E1, NR2E3, NR2F1, NR2F2, NR2F6, NR3C1, NR3C2, NR4A1, NR4A2, NR4A3, NR5A1, NR6A1, NRF1, OTX1, OTX2, PAX3, PAX4, PAX6, PAX7, PDX1, PGR, PHOX2A, PHOX2B, PITX1, PKNOX1, PKNOX2, POU1F1, POU2F1, POU2F2, POU2F3, POU3F1, POU3F2, POU3F3, POU3F4, POU4F1, POU4F2, POU4F3, POU5F1, POU5F1B, POU6F2, PPARA, PPARD, PPARG, PRDM1, PROP1, PRRX1, PRRX2, RARA, RARG, RAX2, REST, RFX1, RFX2, RFX3, RFX5, RFX8, RHOXF1, RORA, RORC, RXRA, RXRB, RXRG, SHOX, SHOX2, SMAD2, SMAR

xxxx

on meanription butionsthe distributions come from 00000000000000000000000000000000000000000000000000000000000000000000000CC1, SOX10, SOX11, SOX14, SOX15, SOX18, SOX2, SOX21, SOX8, SPDEF, SREBF2, SRF, SRY, STAT1, STAT3, STAT4, STAT5A, STAT5B, STAT6, TBP, TBPL2, TBX1, TBX20, TBX21, TCF15, TEF, TFAP4, TFE3, TFEB, TGIF1, TGIF2, TGIF2LX, THRA, THRB, UBP1, UNCX, VAX1, VAX2, VENTX, VSX1, VSX2, YY2, ZBTB33, ZBTB49, ZFHX3, ZKSCAN4, ZNF143, ZNF274, ZNF35, ZNF713, ZNF740, ZNF75A, ZSCAN4 (transcription factors names refer to official symbols according to HUGO Gene Nomenclature Committee).

**TF_disrupt_pval** – P-value of disrupting putative strongest transcription factor binding site

**class** – class label (0 – control, 1 – positive)
