# Supplementary material for: ShapeGTB: the role of local DNA shape in prioritization of functional variants in human promoters with machine learning
Source: PeerJ. 2018 Nov 29;6:e5742. doi: 10.7717/peerj.5742 (PMC6275119; doi:10.7717/peerj.5742)
Supplement: Supplemental Information 5 [file peerj-06-5742-s005.doc]

Supplementary Material 5


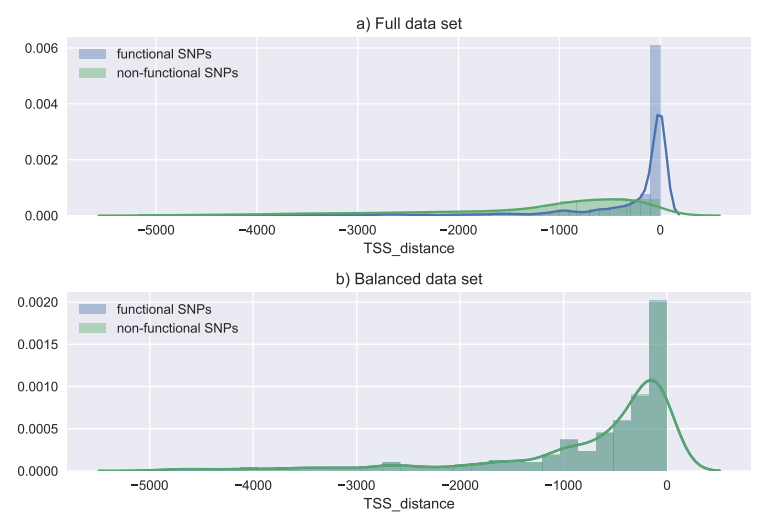
Since variants in the collected data occurred at different locations relative to transcription start sites (TSS) we performed additional analysis to explore what effect TSS balance has on classification performance. We reduced data sets in such a way that the distribution of TSS distance was similar among positive and negative examples. Because of data scarcity ratio of positives to negatives in this dataset became 1:1. The balanced data set contained 884 positive examples and 884 negative examples (reduced training set with balanced TSS contained 692 positives and 654 negatives, reduced test set 230 positives and 192 negatives). Figure S1 presents TSS distance composition for functional and non-functional SNPs in the full data set and TSS-balanced dataset.

Figure S1: Composition of TSS distances in both datasets.

Table S1 presents results obtained in cross validation experiment performed on the TSS-balanced train set with folds determined by chromosomes. The results are very similar to those obtained on the full data set. Obtained average precision (AP) of ShapeGTB classifier trained on TSS-balanced train set and tested on TSS-balanced test set was 0.93, which is the same as one obtained for classifier trained and tested on the full data. These results convince us that performance of ShapeGTB classifier is not influenced by TSS distance composition.

|  | AUC | AUC_std | Accuracy | Accuracy_std | F1 | F1_std | Precision | Precision_std | Recall | Recall_std | size |
| --- | --- | --- | --- | --- | --- | --- | --- | --- | --- | --- | --- |
| All | 0.9450 | 0.0386 | 0.8722 | 0.0554 | 0.8734 | 0.0599 | 0.8478 | 0.0846 | 0.9062 | 0.0650 | 492.0 |
| Best 25 | 0.9238 | 0.0470 | 0.8707 | 0.0462 | 0.8712 | 0.0573 | 0.8438 | 0.0820 | 0.9062 | 0.0652 | 25.0 |
| Sequence | 0.6112 | 0.0899 | 0.5831 | 0.0731 | 0.5841 | 0.0893 | 0.5836 | 0.1155 | 0.5968 | 0.0909 | 52.0 |
| GC content | 0.7582 | 0.0619 | 0.7118 | 0.0490 | 0.7249 | 0.0698 | 0.6837 | 0.0873 | 0.7805 | 0.0879 | 8.0 |
| Shape | 0.5380 | 0.0938 | 0.5273 | 0.0678 | 0.5223 | 0.0896 | 0.5316 | 0.1187 | 0.5262 | 0.0901 | 88.0 |
| Conservation | 0.5059 | 0.0686 | 0.4920 | 0.0605 | 0.4689 | 0.0937 | 0.4944 | 0.1235 | 0.4558 | 0.0880 | 10.0 |
| Transcription factors | 0.5179 | 0.1143 | 0.5197 | 0.0853 | 0.3844 | 0.1244 | 0.5424 | 0.1822 | 0.3068 | 0.1086 | 12.0 |
| Histone modifications | 0.5549 | 0.1248 | 0.5388 | 0.1018 | 0.4858 | 0.1205 | 0.5565 | 0.1322 | 0.4544 | 0.1533 | 38.0 |
| DNase I | 0.4953 | 0.0808 | 0.4690 | 0.0637 | 0.2725 | 0.1275 | 0.5091 | 0.1854 | 0.2329 | 0.1630 | 1.0 |
| Dinucleotide content | 0.5498 | 0.0941 | 0.5351 | 0.0907 | 0.5221 | 0.1109 | 0.5408 | 0.1295 | 0.5237 | 0.1344 | 16.0 |
| TF motifs + TF disruption pval | 0.4880 | 0.0853 | 0.4761 | 0.0779 | 0.2709 | 0.1281 | 0.4547 | 0.2046 | 0.2085 | 0.1139 | 267.0 |
| Sequence + GC content | 0.7706 | 0.0590 | 0.6902 | 0.0691 | 0.6979 | 0.0897 | 0.6748 | 0.1136 | 0.7318 | 0.0881 | 60.0 |
| Shape + GC content | 0.8949 | 0.0506 | 0.8226 | 0.0551 | 0.8257 | 0.0534 | 0.8123 | 0.0833 | 0.8476 | 0.0734 | 96.0 |
| Sequence + GC content + Shape | 0.9616 | 0.0347 | 0.8976 | 0.0399 | 0.9005 | 0.0379 | 0.8715 | 0.0603 | 0.9351 | 0.0461 | 148.0 |
| Sequence + GC content + Shape + TF disruption pval | 0.9624 | 0.0331 | 0.9067 | 0.0455 | 0.9101 | 0.0415 | 0.8779 | 0.0745 | 0.9507 | 0.0509 | 149.0 |
| Sequence + GC content + Transcription factors | 0.7628 | 0.0687 | 0.6830 | 0.0760 | 0.6820 | 0.0991 | 0.6729 | 0.1018 | 0.7034 | 0.1360 | 72.0 |
| Sequence + GC content + Histone modifications | 0.7681 | 0.0567 | 0.7002 | 0.0594 | 0.6994 | 0.0514 | 0.7032 | 0.0770 | 0.7032 | 0.0695 | 98.0 |

Table S1: Performance of XGB classifier trained on different feature combinations.
